# Comparative pilot study of three commercial kits for bacterial DNA extraction from human subgingival biofilm samples collected with a single paper point

**DOI:** 10.1080/20002297.2025.2549035

**Published:** 2025-08-21

**Authors:** Janine Wäge-Recchioni, Renke Perduns, Kirstin Vach, Angela Beckedorf, Joachim Volk, Nadine Schlueter, Ingmar Staufenbiel

**Affiliations:** Department of Conservative Dentistry, Periodontology and Preventive Dentistry, Hannover Medical School, Hannover, Germany

**Keywords:** DNA extraction, subgingival biofilm, paper points, qPCR, periodontal research

## Abstract

**Objective:**

In periodontal research, subgingival biofilm samples are typically collected using sterile paper points and pooled for molecular analyses. Streamlining this process by using a single paper point for molecular analysis could simplify sample collection and allow additional paper points to be used for other investigations. This pilot study evaluated the performance of three commercial DNA extraction kits for analysing small sample volumes (<10 µL).

**Methods:**

Samples were collected from six participants, each contributing 18 paper points from both healthy and periodontitis-affected sites. Bacterial and human DNA yields were quantified using fluorometric measurements combined with qPCR, employing universal 16S primers for bacterial DNA and human-specific GAPDH primers.

**Results:**

Among the tested kits, the DNeasy Blood and Tissue Kit demonstrated the highest efficiency, yielding significantly more total dsDNA in samples from healthy sites compared to both other kits and in samples from periodontitis-affected sites compared to one kit. Bacterial DNA yields were also significantly higher with the DNeasy Kit compared to one of the other kits in both health conditions.

**Conclusion:**

These results suggest that one paper point is sufficient to extract DNA for subsequent bacterial analyses and that the DNeasy Blood and Tissue Kit appears to be the most efficient among the three tested kits.

## Introduction

The human oral cavity harbours a highly diverse microbiome, comprising bacteria, microeukaryotes, archaea, and viruses [1,2]. Distinct microenvironments, such as dental enamel, keratinized surfaces, and soft tissues, shape microbial communities through factors like oxygen gradients, nutrient availability, and salivary influence [1]. While most microorganisms are distributed across the oral cavity via saliva, their abundances reflect niche-specific adaptations [3]. Subgingival biofilms are particularly relevant for periodontal health, as microbial composition and function are linked to diseases like gingivitis and periodontitis [4,5]. Accurate characterization of these communities is crucial for understanding microbial ecology and developing targeted therapies [6].

Sterile paper points are commonly used for subgingival biofilm sampling, providing results mostly comparable to curettes [7]. In research, multiple paper points are often pooled, though this is not always feasible when investigating diverse questions. Using a single paper point for sampling offers several advantages compared to pooling multiple points from the same or different sampling locations. It reduces the amount of material costs, minimises patient discomfort, and shortens the sampling time. Furthermore, utilising a single paper point for DNA analyses preserves a greater amount of biofilm within the same periodontal pocket, allowing for subsequent sampling to assess other parameters. This approach provides a more accurate representation of the microbial community at a specific site. In contrast, pooling multiple points from different pockets may lead to dilution or mixing of bacterial populations from distinct microenvironments. Therefore, single-point sampling can enhance the accuracy and specificity of microbial analyses in periodontal research, particularly when addressing targeted research questions. Various commercial DNA extraction kits employ different methodologies that may influence DNA yield, purity, and integrity.

This pilot study, conducted with a limited number of participants, compares three DNA extraction kits – NucleoSpin Tissue Mini (MACHEREY-NAGEL), ZymoBIOMICS DNA Miniprep (ZYMO RESEARCH), and DNeasy Blood and Tissue (QIAGEN) – for their efficiency in isolating microbial gDNA from single paper point samples collected from individual periodontal pockets. The findings will support the development of a subsequent large-scale study designed to investigate multiple parameters from individual periodontal pockets. These kits were chosen to reflect a broad spectrum of lysis approaches: enzymatic, chemical, mechanical, or combinations of these, and based on their prior use or manufacturer recommendations for oral microbial applications (e.g. [8–11]). Kits were evaluated based on gDNA yield, integrity, purity, bacterial DNA recovery, PCR inhibitors, cost, and processing time. The results aid in selecting an optimal extraction method for microbial analysis of smallest quantities from subgingival biofilms. To our knowledge, no prior study has systematically assessed DNA extraction kit performance using such small subgingival biofilm sample volumes.

## Material and methods

This study was approved by the Institutional Review Board (Ethics Committee of Hannover Medical School) (approval no. 11256_BO_K_2024). The study was conducted at the Hannover Medical School, Department of Conservative Dentistry, Periodontology and Preventive Dentistry, Hannover, Germany. Participants were recruited during regular dental check-ups and were undergoing supportive periodontal therapy at the time of sample collection. Details on the classification of periodontitis (stage, grade, and probing pocket depth at the sampling point) are provided in Supplemental Table S1. The mean age (±SD) of the participants (5 males, 1 female) was 55.3 (±9.0) years (range 39–62 years). All participants were generally healthy and non-smokers. One participant was receiving antihypertensive medication with an angiotensin-converting enzyme (ACE) inhibitor. Inclusion criteria were written informed consent, legal age and presence of healthy and periodontally affected tooth sites. Exclusion criteria for the study were any systemic antibiotic treatment during the last 12 weeks before sample collection, pregnancy and breastfeeding.

### Paper point samples

Subgingival biofilm samples, including a mixture of crevicular fluid, biofilm, and bacterial content, were collected from six participants at two periodontally healthy sites (pocket depth <4 mm, no bleeding on probing) and two periodontitis-affected sites (pocket depth between 4 mm and 12 mm, bleeding and nonbleeding on probing) by inserting a sterile paper point (VDW Dental, 29 mm, size 50) into the periodontal pocket for 15 s. Subgingival biofilm samples were collected from periodontitis-affected pockets, which generally exhibit high microbial biomass, and from healthy periodontal pockets, typically associated with low biofilm accumulation. A total of 108 samples were collected, with nine paper points per health condition from each participant. The samples were distributed among the kits, ensuring that each kit received an equal number of samples from each participant (see Supplemental Table S2). Before sampling, the teeth were isolated with cotton rolls, and the supragingival surface was gently dried with an air syringe to avoid contamination [12,13]. Afterwards, the paper points were transferred into a sterile 1.5 mL tube and rapidly frozen in liquid nitrogen. All samples were stored at −80°C until further processing.

### Cell lysis and DNA isolation

The following commercially available kits were used to extract DNA from single paper points according to the manufacturer’s instructions: (1) NucleoSpin Tissue, Mini Kit for DNA from cells and tissue (MACHEREY‑NAGEL, Düren, Germany), (2) ZymoBIOMICS DNA Miniprep Kit (ZYMO RESEARCH, Freiburg, Germany) and (3) DNeasy Blood & Tissue Kit (QIAGEN, Hilden, Germany) (Table 1). Deviations from the protocol and choices concerning alternative or adjustable steps are described below.

**Table 1.** DNA extraction kits compared in this study.

Name of kit	Manufacturer	Cell lysis	Kit name abbreviation	Elution volume (µL)	Price* per extraction (€)	Time per extraction (min)
NucleoSpin Tissue Mini	MACHEREY ‑NAGEL	enzymatic and chemical lysis (proteinase K/SDS solution)	MN	60–100	3.48	~90
ZymoBIOMICS DNA Miniprep	ZYMO RESEARCH	mechanical lysis by bead beating and lysis solution (contains EDTA)	ZB	50–100	6.51	~120
DNeasy Blood & Tissue	QIAGEN	enzymatic and chemical lysis (Tris-Cl, sodium EDTA, Triton X-100, lysoszme)	QB	100–200	4.48	~150

*Netto price for a kit size of 50 preparations.

#### MACHEREY‑NAGEL kit protocol

To pre-lyse the sample, the manufacturer’s instructions were followed as described in section 6.16 - Support protocol for purification of genomic DNA from dental swabs. To separate the lysis solution from the paper point, method C was used, whereby as much lysate as possible was transferred with a pipette. For the elution step, 100 µL elution buffer BE was used.

#### ZYMO RESEARCH kit protocol

Mechanical lysis by bead beating with ultra-high density BashingBeads and lysis solution was performed using an IKA VXR Vibrax vortex shaker (IKA-Werke GmbH & Co. KG, Staufen, Germany), by vortexing on maximum speed for 1 min, followed by a 5-min pause. This procedure was repeated five times. For the elution step, 100 µL DNase/RNase free water was used.

#### QIAGEN kit protocol

The casing (pipe‐in‐pipe) method according to Lu et al. [14] was used to wash the sample material out of the paper point: 1 mL of nuclease-free water (Ambion by Thermo Fisher Scientific, Schwerte, Germany) and 12 glass beads (1.7–2.1 mm, Carl Roth, Karlsruhe, Germany) were added to the paper point in a 1.5 mL tube and shaken at 14,000 rpm for 5 min. The tube was pierced at the bottom and placed into a 5 mL centrifuge tube. After centrifugation at 4,000 × g for 1 min, the flow-through was transferred from the 5 mL tube into a new 1.5 mL centrifuge tube and pelleted for 15 min at 10,000 × g. The obtained pellet was resuspended in 180 µL enzymatic lysis buffer, and the protocol for pretreatment for Gram-positive bacteria followed by the standard protocol for ‘Purification of Total DNA from Animal Tissues’ were applied. 100 µL Buffer AE was used to elute the DNA.

### Total DNA and double-stranded DNA quantification

Total DNA yield (ng per paper point) was analysed by UV absorption at 260 nm using a NanoDrop TM 1000 spectrophotometer (Thermo Fisher Scientific, Schwerte, Germany). Additionally, the yield of double-stranded DNA (dsDNA) was determined using the Invitrogen Qubit 2.0 fluorometer (Thermo Fisher Scientific, Schwerte, Germany) following the manufactures instructions. The Qubit assay utilizes a target-specific fluorescent dye that exhibit fluorescence exclusively upon binding to dsDNA. The measurements were performed by the Research Core Unit Genomics at the Hannover Medical School.

### Quantification of bacterial and coextracted human DNA by qPCR

The bacterial and human DNA yields were quantified by quantitative real-time PCR (qPCR) on a CFX96 Real-Time PCR Detection System (Bio-Rad, Feldkirchen, Germany), using universal primer pairs specific for the bacterial 16S rRNA gene ([15]; forward primer: 5′-TCCTACGGGAGGCAGCAGT-3; reverse primer: 5’- GGACTACCAGGGTATCTAATCCTGTT −3“) and the human GAPDH gene ([16]; forward primer: 5′-TGCACCACCAACTGCTTAGC-3”, reverse primer: 5′-GGCATGGACTGTGGTCATGAG-3’). For the qPCR reactions, a final volume of 10 µL containing 5 µL of TB Green Premix Ex Taq (TaKaRa, Cologne, Germany), 4 µL template DNA and 1 µL primer with a final concentration of 500 nM was used. Cycling conditions: 95°C for 30 s, followed by 40 cycles of 95°C for 5 s and 60°C for 30 s and a step to record the melting curve at the end of the run (60°C for 5 s, 95°C for 5 s). To determine potential contamination, a control lacking DNA template was included in the qPCR analysis. Melting curves and the size of the PCR products on agarose gels were analysed to check for primer specificity and the absence of primer dimers. The amplification efficiency of each primer pair was validated via serial DNA dilutions. Based on the resulting threshold (Ct) values for human and bacterial DNA, the relative abundance of bacterial versus human DNA was calculated using the delta Ct method. These ratios were then applied to the total dsDNA concentration, previously quantified by Qubit fluorometer, to determine the relative and absolute contributions of each DNA type to the total amount of DNA.

### Evaluation of DNA purity and integrity

The DNA purity [absorbance ratio at A_260_/A_280_] was analysed using a NanoDrop TM 1000 spectrophotometer (Thermo Fisher Scientific, Schwerte, Germany). An A_260_/A_280_ ratio of 1.8–2.0 is considered as pure DNA. Ratios >2.0 indicate RNA contamination and ratios <1.8 indicate contaminations with residual phenol, protein, or other reagents.

The DNA integrity was assessed by calculating the ratio of extraction yields obtained fluorometrically to quantify dsDNA, compared to spectrophotometric measurements, which determines the UV absorption at 260 nm (‘NanoDrop-to-Qubit quotient’). The latter reflects the concentration of all nucleic acids (DNA and RNA), along with their nucleotides and other contaminants [17]. A quotient near 1.0 indicates high quality, intact DNA, while higher quotients suggest possible degradation or contamination.

### Determination of PCR inhibitors

To asses DNA extracts for potential PCR inhibitors, a combination of spectrophotometric analysis and functional testing by qPCR was employed. The A_260_/A_280_ ratio serves as an indicator of contaminant presence, with ratios below 1.8 typically indicating contamination by phenol or other reagents that may interfere with enzymatic activity during PCR. Ratios exceeding 2.0 suggest RNA contamination, potentially impacting downstream applications.

Additionally, undiluted and 10-fold diluted DNA samples were analysed by real-time qPCR to identify inhibitors such as chemical residues from the extraction kit. At 100% amplification efficiency, a theoretical ΔCt of 3.32 is expected between undiluted and diluted samples. PCR inhibitors would lower the ΔCt between two sample dilutions because they may interfere with different steps of a PCR, e.g. disruption of the annealing of the primers to the DNA template or inhibiting the activity of the DNA polymerase [18]. DNA samples were considered free of inhibitors if the observed ΔCt was within 3.32 ± 0.5.

### Statistical analysis

For descriptive analysis median, mean values and standard deviations were calculated. For graphical presentation and testing, the nucleic acid values were log10 transformed. Due to the skewed distribution of the data, Wilcoxon signed rank tests were used for group comparisons. As this was an exploratory study, no corrections for multiple testing were made. The significance level was set to 5%. All computations were done with STATA (version 17.0, College Station, TX, USA).

## Results

### Total genomic DNA and dsDNA yield

**Figure 1.**
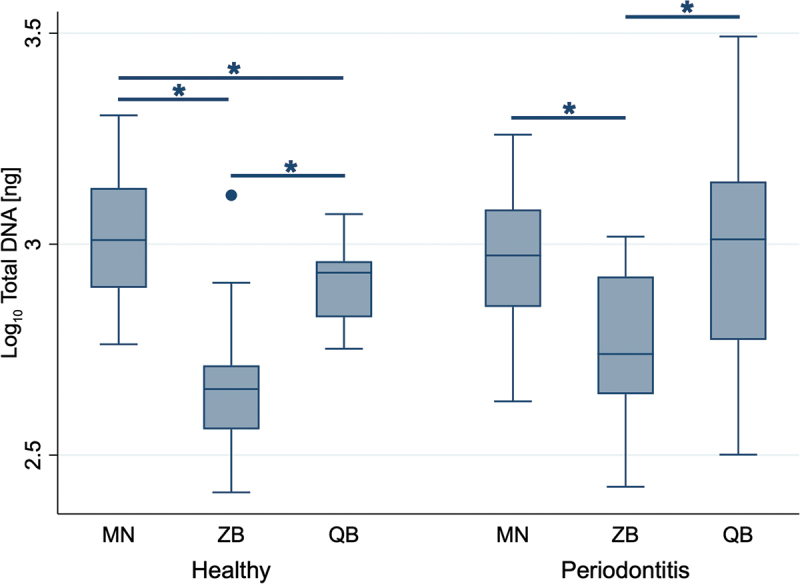
Total DNA extracted from single paper points. The total DNA was quantified by spectrophotometric measurement. Data represent 18 samples per health condition (periodontal healthy and periodontitis-affected sites) collected from six participants for each of the three kits, shown on a logarithmic scale. MN: NucleoSpin Tissue kit, MACHEREY‑NAGEL, ZB: ZymoBIOMICS DNA Miniprep kit, ZYMO RESEARCH, QB: DNeasy Blood & Tissue kit, QIAGEN. Significant differences (Wilcoxon signed-rank test) are indicated by an asterisk (*).

The spectrophotometric analysis of total gDNA revealed that the NucleoSpin Tissue Mini (MN) kit achieved the highest nucleic acid yields, with values ranging from 579 ng to 2020 ng in healthy samples and 424 ng to 1818 ng in samples from periodontitis-affected sites. Statistical analysis confirmed that the MN kit resulted in significantly higher total nucleic acid yields than both the ZymoBIOMICS DNA Miniprep (ZB) (*p* = 0.028) and DNeasy Blood & Tissue (QB) (*p* = 0.046) kit in healthy samples (Figure 1). Similarly, in periodontitis-affected samples, the MN kit yielded significantly higher amounts of nucleic acid compared to the ZB kit (*p* = 0.028), whereas no significant difference was observed between the MN and QB kit (*p* = 0.600). The ZB kit consistently achieved the significantly lowest DNA yields across both health conditions, ranging from 258 ng to 1306 ng in healthy samples and 266 ng to 1042 ng in periodontitis-affected samples. The QB kit resulted in the widest range of nucleic acid yields in samples from periodontitis-affected sites, with values ranging from 317 ng to 3107 ng (Figure 1).

**Figure 2.**
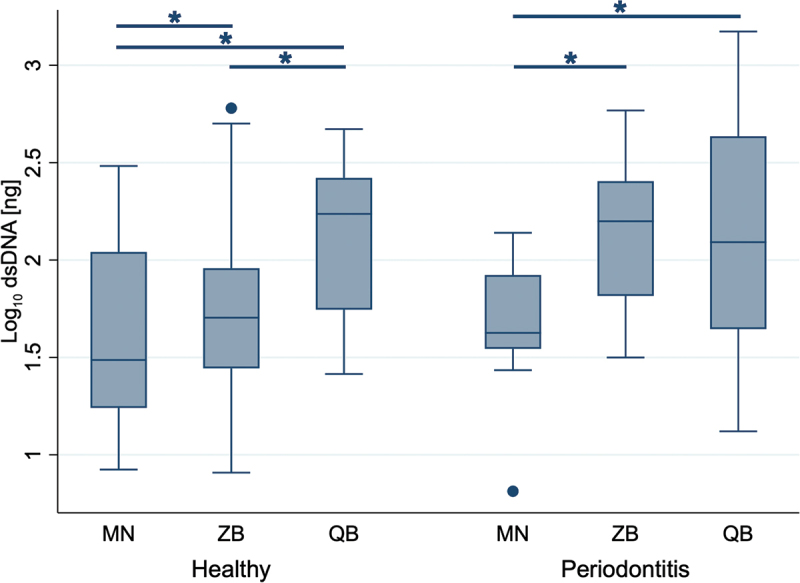
dsDNA yields extracted from single paper points. The dsDNA yields were quantified fluormetrically. Data represent 18 samples per health condition (periodontal healthy and periodontitis-affected sites) collected from six participants for each of the three kits, shown on a logarithmic scale. MN: NucleoSpin Tissue kit, MACHEREY‑NAGEL, ZB: ZymoBIOMICS DNA Miniprep kit, ZYMO RESEARCH, QB: DNeasy Blood & Tissue kit, QIAGEN. Significant differences (Wilcoxon signed-rank test) are indicated by an asterisk (*).

Double-stranded DNA (dsDNA) yields varied significantly between the three extraction kits (Figure 2). The MN kit resulted moderate yields for both healthy (8.4 ng−304 ng) and periodontitis-affected (6.5 ng−138 ng) samples, with statistically lower dsDNA amounts than those obtained with the QB kit in both sample types (*p* = 0.028 for healthy, *p* = 0.046 for periodontitis-affected) and the ZB kit (*p* = 0.028 for both). The amount of isolated dsDNA with the ZB kit showed a high variability, particularly in healthy samples (8.1 ng to 602 ng). Furthermore, the statistical analysis revealed that in healthy samples, dsDNA yields from the QB kit were also significantly higher than those from the ZB kit (*p* = 0.028) (Figure 2). In general, the QB kit exhibited the widest range of dsDNA yields, particularly in periodontitis-affected samples (13.2 ng to 1490 ng).

### Yield of amplifiable bacterial DNA

**Figure 3.**
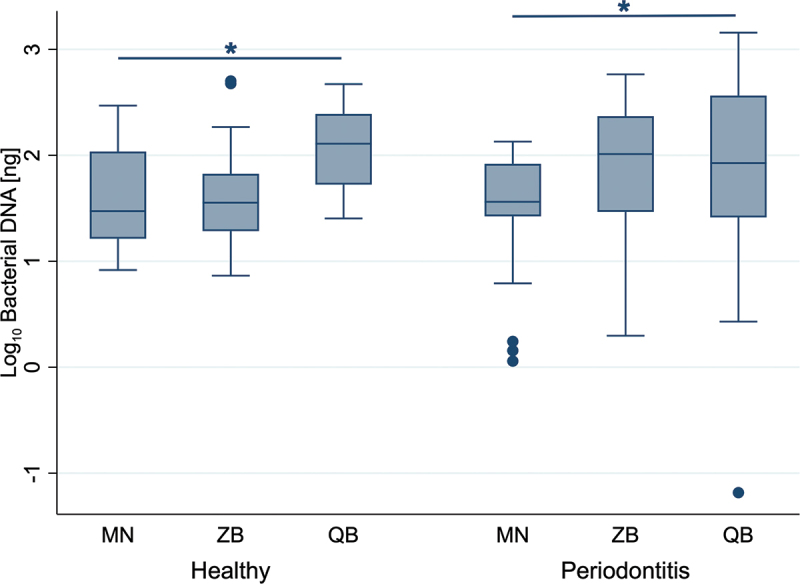
Yield of bacterial DNA extracted from single paper points. The share of bacterial DNA was calculated by applying the ratio between human and bacterial DNA, determined by qPCR, to the total amount of dsDNA, quantified by fluorometric measurements. Data represent 18 samples from periodontal healthy and periodontitis-affected sites across six participants, shown on a logarithmic scale. MN: NucleoSpin Tissue kit, MACHEREY‑NAGEL, ZB: ZymoBIOMICS DNA Miniprep kit, ZYMO RESEARCH, QB: DNeasy Blood & Tissue kit, QIAGEN. Significant differences (Wilcoxon signed-rank test) are indicated by an asterisk (*).

Bacterial DNA yields across the three kits showed considerable variability (Figure 3). The MN kit yielded bacterial DNA amounts ranging from 8.27 to 294.53 ng in healthy samples, and 1.14 to 134.66 ng in samples from periodontitis-affected sites. The ZB kit resulted in bacterial DNA amounts of 7.31 to 503.07 ng in healthy individuals and 1.98 to 581.48 ng in periodontitis-affected samples. The QB kit achieved bacterial DNA amounts of 25.38 to 470 ng in healthy individuals and the widest range in samples from periodontitis-affected sides of all three kits with 0.07 to 1438.29 ng. The Wilcoxon signed-rank test showed significant differences between some kits. The QB kit yielded in significantly higher bacterial amounts compared to the MN kit for healthy as well as periodontitis-affected samples (*p* = 0.028 and *p* = 0.046). No significant differences were observed between the other kits and sample groups (*p* > 0.05).

### DNA purity

**Figure 4.**
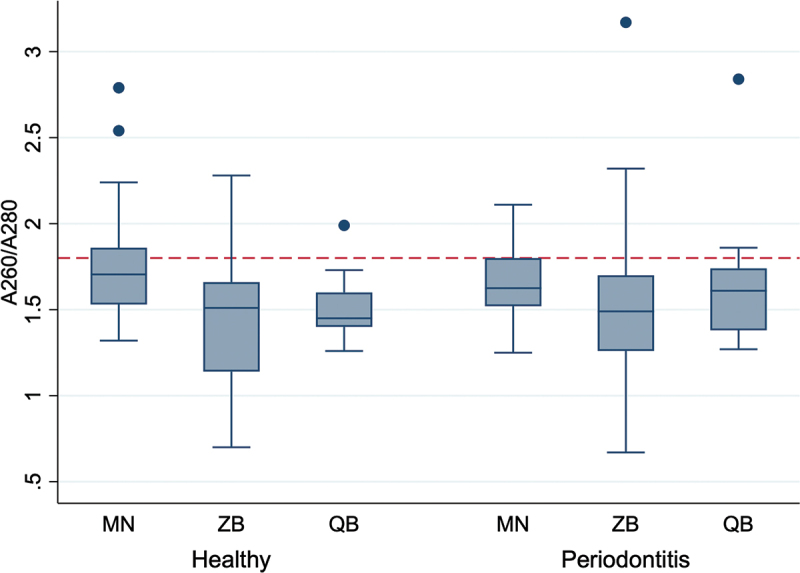
A_260_/A_280_ ratios of DNA extracted from single paper points. The A_260_/A_280_ ratios were determined using spectrophotometric analysis. Data represent 18 samples per health condition (periodontal healthy and periodontitis-affected sites) collected from six participants for each of the three kits, except for the ZB healthy group, where one sample was excluded. The data is shown on a logarithmic scale. The red dashed line shows the ideal 260/280 ratio for pure DNA (~1.8). MN: NucleoSpin Tissue kit, MACHEREY‑NAGEL, ZB: ZymoBIOMICS DNA Miniprep kit, ZYMO RESEARCH, QB: DNeasy Blood & Tissue kit, QIAGEN.

The A_260_/A_280_ ratios obtained showed differences in DNA purity and consistency across the three kits (Figure 4). The MN kit resulted in DNA samples with A_260_/A_280_ ratios closer to the ideal value of 1.8 (healthy: 1.77 ± 0.38, periodontitis-affected: 1.64 ± 0.22), compared to those obtained with the other two kits (ZB: healthy: 1.43 ± 0.45, periodontitis-affected: 1.56 ± 0.54; QB: healthy: 1.50 ± 0.17, periodontitis-affected: 1.65 ± 0.34). In contrast, DNA obtained with the ZB and QB kits had many A_260_/A_280_ ratios below the 1.8 threshold, and the ZB kit exhibited substantial variability in these values.

### DNA integrity

**Figure 5.**
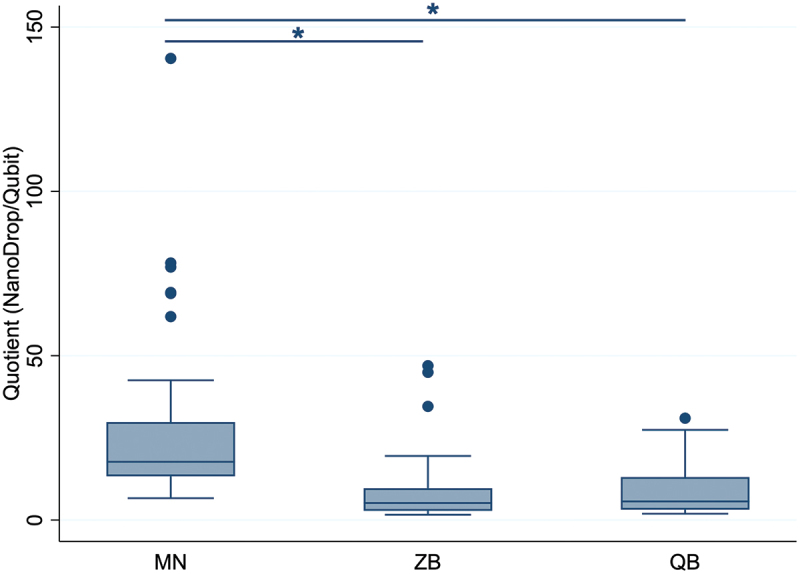
DNA integrity based on the ratio of total nucleic acid and dsDNA concentration. Data represent 36 samples per extraction kit. MN: NucleoSpin Tissue kit, MACHEREY‑NAGEL, ZB: ZymoBIOMICS DNA Miniprep kit, ZYMO RESEARCH, QB: DNeasy Blood & Tissue kit, QIAGEN. Significant differences (mixed-effects model) are indicated by an asterisk (*).

The DNA integrity differed significantly between the kits (Figure 5). DNA samples extracted with the MN kit (mean value = 29.20 ± 27.26) showed significantly higher quotients (both *p* < 0.001) and therefore a lower integrity than DNA samples extracted with ZB (mean value = 9.47 ± 11.03) and QB kits (mean value = 9.12 ± 8.23). No significant difference in the DNA integrity was found between DNA isolated with the ZB and QB kits (*p* = 0.261).

### Determination of PCR inhibitors

In order to assess the presence of PCR inhibitors, the A_260_/A_280_ ratio were examined. The A_260_/A_280_ ratio revealed notable differences among the extraction kits. The MN kit resulted in DNA samples with A_260_/A_280_ ratio closer to the ideal value of 1.8, however there were also samples with lower or higher ratios (healthy: 1.77 ± 0.38, periodontitis-affected: 1.64 ± 0.22). DNA samples processed with the ZB kit showed a high variability in the A_260_/A_280_ ratio with the majority of samples having ratios under 1.8 (healthy: 1.43 ± 0.45, periodontitis-affected: 1.56 ± 0.54). DNA samples from the QB kit resulted relatively consistently low A_260_/A_280_ ratios (healthy: 1.50 ± 0.17, periodontitis-affected: 1.65 ± 0.34).

Furthermore, 10-fold dilutions of the DNA extracts were analysed by real-time qPCR. Results indicate minor inhibition of the qPCR in five of the 72 DNA samples extracted with the ZB kit (No. 19-16S, 28-16S, 33-16S, 9-GAPDH and 16-GAPDH), where Ct value differences remained below 3.32 ± 0.5, ranging from 2.3 to 2.7. Similarly, inhibition was observed in five samples processed with the QB kit (No. 19-16S, 34-16S, 36-16S, 19-GAPDH, and 36-GAPDH), with Ct differences ranging from 2.2 to 2.6. In contrast, the MN kit exhibited the least inhibition, with only one sample (No. 5) affected when using the GAPDH primer (Supplemental Table S3).

## Discussion

The findings of the present study contribute valuable information about bacterial DNA extraction kits in dental and periodontal research, particularly when extracting from very small amounts of subgingival biofilm samples. We could demonstrate that a single paper point can yield sufficient DNA for further downstream application such as quantitative PCR, overcoming the need of pooling multiple paper points depending on the application. Reducing the number of paper points not only makes the collection process more convenient, but also allows to use additional paper points to explore a broader range of parameters, e.g. inflammatory markers, from the very same periodontal pocket. Additionally, we compared the performance of three kits to isolate bacterial DNA to facilitate the selection of the most suitable kit for future studies in the field. The QB Kit demonstrated overall the best results, yielding high amounts of total DNA, dsDNA, and bacterial DNA across the tested conditions.

In general, subgingival biofilms play a crucial role in understanding the microbial ecology of the oral cavity and the pathogenesis of periodontal diseases [5]. They provide a protective function in maintaining a balanced ecosystem in the oral cavity [19]. On the other hand, disruptions in the balance between different microbes can cause chronic inflammatory reactions, leading to gingivitis or periodontitis [20]. It is known that periodontitis is linked to systemic diseases such as cardiovascular conditions or diabetes, which make precise microbial profiling essential to explore broader health implications [21].

Traditional DNA extraction methods, such as phenol-chloroform extraction, are very time consuming and challenging to standardise across different operators and sample conditions. Additionally, these techniques involve the use of hazardous organic solvents [22]. In order to standardise the DNA extraction, commercial kits to isolate microbial DNA are very useful, especially for projects that require high numbers of samples. To our knowledge, no study has yet compared commercial DNA extraction kits for subgingival biofilm samples or scientifically tested whether single paper points are sufficient to isolate enough DNA for prevalent downstream applications, like qPCR analyses. Our comparative analysis included three commercial DNA extraction kits employing enzymatic/chemical lysis (MN, QB) and, for ZB, additional mechanical lysis by bead beating (Table 1).

Our initial assessment of total DNA yield indicated that the MN kit resulted in the highest yields overall, particularly in healthy samples, while in periodontitis-affected samples, it significantly outperformed the ZB kit but not the QB kit (Figure 1). However, fluorometric quantification showed that the MN kit achieved considerably lower dsDNA yields compared to the ZB and QB kits, highlighting the tendency of spectrophotometry to overestimate DNA yield by detecting all nucleic acids and nucleotides [23].

As intact dsDNA is required for many downstream applications, it is essential to specifically quantify dsDNA. Overall, we found that the isolation of dsDNA was more efficient with the QB kit, which is using enzymatic and chemical cell lysis and no additional bead-beating step like the ZB kit (Figure 2). Although mechanical disruption with bead-beating is recommended to break microbial cell walls with thick peptidoglycan layers [24,25], some DNA-containing solution may have remained trapped between the beads during the transfer step. In addition, the ZB kit’s use of the Zymo-Spin III-F Filter may have further reduced the overall yield. Another factor that may influence DNA yield is the incubation time during enzymatic lysis, which varies among the kits. The QB kit has the longest total incubation duration at 60 min, compared to 30 min for the MN and 25 min for the ZB kit. This extended time might enhance the lysis efficiency of resilient microorganisms like gram-positive bacteria [26], potentially contributing to higher dsDNA yields.

However, notable variability in DNA yields was observed across kits and samples. Gingival bleeding influences DNA yields and composition, which may introduce host DNA and impact both yield and downstream microbiome analysis by affecting microbial diversity, altering the detected composition, and limiting the comparability between samples and extraction methods. Therefore, it is important to recognise that the measured DNA includes not only microbial DNA but also co-extracted human DNA and heavily fragmented DNA. To accurately compare the bacterial DNA yield across the extraction kits, qPCR analyses targeting the bacterial 16S rRNA gene and the human GAPDH gene were performed, revealing that all tested kits extracted both bacterial and human DNA.

Especially, in samples from periodontitis-affected sites, human DNA contamination is often unavoidable due to the attraction of immune cells to the inflamed tissue and through bleeding. Human DNA can also originate from epithelial cells present in gingival crevicular fluid. Human DNA contamination poses a significant challenge for next-generation sequencing (NGS) technologies, such as metagenome analyses, which sequence all DNA present in the sample [27].

Among the tested kits, the QB kit yielded significantly higher amounts of bacterial DNA than the MN kit in both healthy and periodontitis-affected samples, whereas no significant difference was observed between the QB and ZB kits (Figure 3). These findings highlight the QB kit as a suitable option but suggest that the ZB kit may perform comparably.

Another factor for the successful extraction of bacterial DNA is the quality of the extracted DNA, which heavily affects the reliability of downstream applications. Our results indicated that DNA extracted using the MN kit exhibited a significantly higher NanoDrop-to-Qubit quotient compared to the ZB and QB kits, implying lower DNA integrity. No significant difference was observed between the ratios of the DNA isolated with the ZB and QB kits, indicating that both kits perform similarly with respect to the integrity of the extracted DNA. These findings align with previous studies that reported variations in DNA yield and purity across different extraction methods like manual phenol-chloroform extraction method and commercially available kits [28,29]. Importantly, while DNA yield can vary substantially between protocols, multiple studies demonstrate that the overall bacterial and fungal community composition remains largely stable across extraction methods [30,31]. In contrast, an optimised sample preparation method using ceramic beads significantly improved both DNA yield and microbial diversity in subgingival plaque from periodontitis patients compared to the standard approach [32].

The A_260_/A_280_ ratio provides insights into the purity of DNA, particularly concerning protein contamination. In our study, DNA extracted using the MN kit exhibited A_260_/A_280_ ratios close to 1.8, suggesting minimal protein contamination. The QB and ZB kits yielded lower A_260_/A_280_ ratios, with the ZB kit showing a high variability between samples. These observations imply that while the MN kit effectively minimizes protein contamination, it may not preserve DNA integrity as efficiently, given the higher NanoDrop-to-Qubit quotients observed. In contrast, the lower and more variable A_260_/A_280_ ratios associated with the QB and ZB kits suggest the opposite: Increased contamination with protein or other impurities but a better preservation of DNA integrity.

To evaluate the presence of inhibitors in the DNA extracts, A_260_/A_280_ ratios were examined. All tested kits showed a tendency for A_260_/A_280_ ratios below 1.8, indicating that residual reagents may not have been fully removed (Figure 4). However, spectrophotometric readings at concentrations below 10 ng/µL are often unreliable due to a poor signal-to-noise ratio. This limitation may partly explain the suboptimal ratios observed, as many samples had low DNA concentrations. This outcome is not surprising, given that the extractions were performed from a single paper point, which inherently yields limited material.

While purity ratios are valuable indicators of sample quality, a better measure of DNA quality lies in its functionality for the intended downstream application. Solvents such as ethanol, frequently used in commercial DNA extraction kits to precipitate DNA, are known inhibitors of DNA polymerases, which are critical for the success of PCR reactions [18]. The presence of these inhibitors can therefore significantly affect PCR accuracy, potentially leading to false-negative or, at least, imprecise results. To further evaluate the effectiveness of the commercial kits in removing these inhibitors, this study assessed the presence of PCR inhibitors in the DNA extracts using qPCR. A 10-fold dilution of the DNA was prepared and analysed with primers targeting the bacterial 16S rRNA and human GAPDH genes. This approach takes advantage of the fact that dilution of inhibitors increases the efficiency of the PCR. With the ZB kit, different samples were inhibited for each primer. In contrast, with the QB kit, two samples showed inhibition with both primers. It is not uncommon for different primers to be affected by inhibitors to varying degrees, as the specificity and amplification efficiency can vary depending on the primer and target sequence. Some primers or target sequences may be more resilient to inhibitors than others. For example, a particular primer design may exhibit a higher affinity for the template DNA, making the PCR more efficient even in the presence of inhibitors [33]. The present findings suggest that the tested commercial kits are generally effective at removing most PCR inhibitors. Nevertheless, the presence of inhibitors remains a critical factor, not only for PCR but also for other molecular applications and downstream analyses.

Gingival crevicular fluid composition is modulated by several factors, including gingival inflammation, mechanical stimulation (e.g. mastication, probing), circadian rhythm, hormonal status (e.g. ovulation, contraceptive use), smoking, and recent dental procedures [34]. Additionally, hormonal fluctuations in women are known to affect gingival crevicular fluid flow and microbial composition, potentially impacting DNA yield [35]. Given the inclusion of only one female and five male participants in this pilot study, potential gender-related variability in the DNA extraction from subgingival biofilm samples cannot be excluded, although the extraction methodology is technically gender-independent. To assess effects of these variables on the DNA extraction from subgingival biofilm samples a large-scale study is required. Furthermore, the bacterial composition of the biofilm should also be investigated in future studies, particularly with regard to the influence of the extraction method and the number of paper points used per sample. In addition, the use of a mock community could help to more precisely assess and compare the extraction efficiency of different kits under defined conditions.

## Conclusions

In conclusion, this pilot study, which included samples from six participants, demonstrates that all tested kits successfully extracted bacterial DNA from very small sample quantities, with differences in yield and quality. The QB kit performed best, providing the highest dsDNA and bacterial DNA yields at moderate costs. While the small sample size and varying sample quantities may influence the starting material, it reflects actual clinical conditions. Overall, the findings offer valuable insights and a good foundation for future research, which should include more comprehensive analyses, particularly sequencing-based approaches.

## Supplementary Material

Waege_Recchioni_Supplemental_Material_clean.docx
