# Supplementary material for: Comparative pilot study of three commercial kits for bacterial DNA extraction from human subgingival biofilm samples collected with a single paper point
Source: J Oral Microbiol. 2025 Aug 21;17(1):2549035. doi: 10.1080/20002297.2025.2549035 (PMC12372513; doi:10.1080/20002297.2025.2549035)
Supplement: Waege_Recchioni_Supplemental_Material_clean.docx [file ZJOM_A_2549035_SM3558.docx]

**Supplemental Material**

This file contains:

1. Supplemental Table 1

2. Supplemental Table 2

3. Supplemental Table 3

**Supplemental Material**

**Supplemental Table 1** Classification of periodontitis according to Tonetti et al. (2018) and probing pocket depth at the sampling points in the six participants.

| **Participant** | **Stage** | **Grade** | **Probing Pocket Depth at Sampling Points (mm)** |
| --- | --- | --- | --- |
| Participant 1 | III | C | 4, 4, 6, 5, 4, 5, 4, 4, 6 |
| Participant 2 | III | B | 5, 5, 4, 4, 4, 4, 6, 4, 5 |
| Participant 3 | IV | C | 6, 6, 6, 5, 7, 6, 5, 5, 6 |
| Participant 4 | IV | C | 4, 4, 10, 6, 4, 5, 6, 5, 8 |
| Participant 5 | IV | C | 6, 6, 6, 6, 6, 6, 6, 6, 6 |
| Participant 6 | IV | C | 8, 5, 6, 5, 6, 8, 4, 6, 7 |

**Supplemental Table 2** Distribution of the nine paper points per participant and health condition across the three extraction kits. The numbers one to nine represent the order of sample collection. MN: NucleoSpin Tissue Kit, MACHEREY-NAGEL; ZB: ZymoBIOMICS DNA Miniprep Kit, ZYMO RESEARCH; QB: DNeasy Blood & Tissue Kit, QIAGEN.

| **Sample** | **MN** | **ZB** | **QB** |
| --- | --- | --- | --- |
| Participant 1 - healthy | 1 - 3 | 4 - 6 | 7 - 9 |
| Participant 2 - healthy | 7 - 9 | 1 - 3 | 4 - 6 |
| Participant 3 - healthy | 4 - 6 | 7 - 9 | 1 - 3 |
| Participant 4 - healthy | 1 - 3 | 4 - 6 | 7 - 9 |
| Participant 5 - healthy | 7 - 9 | 1 - 3 | 4 - 6 |
| Participant 6 - healthy | 4 - 6 | 7 - 9 | 1 - 3 |
| Participant 1 - periodontitis | 1 - 3 | 4 - 6 | 7 - 9 |
| Participant 2 - periodontitis | 7 - 9 | 1 - 3 | 4 - 6 |
| Participant 3 - periodontitis | 4 - 6 | 7 - 9 | 1 - 3 |
| Participant 4 - periodontitis | 1 - 3 | 4 - 6 | 7 - 9 |
| Participant 5 - periodontitis | 7 - 9 | 1 - 3 | 4 - 6 |
| Participant 6 - periodontitis | 4 - 6 | 7 - 9 | 1 - 3 |

**Supplemental Table 3** Assessment of PCR inhibition in DNA extracts. ΔCt analysis of undiluted and 10-fold diluted samples using 16S and GAPDH primers. MN: NucleoSpin Tissue Kit, MACHEREY‑NAGEL; ZB: ZymoBIOMICS DNA Miniprep Kit, ZYMO RESEARCH; QB: DNeasy Blood & Tissue Kit, QIAGEN.

|  | **MN** | | | **ZB** | | | **QB** | | |
| --- | --- | --- | --- | --- | --- | --- | --- | --- | --- |
| **Samples** | **Ct undil** | **Ct 10-fold dil** | **∆Ct (10-fold dil-undil)** | **Ct undil** | **Ct 10-fold dil** | **∆Ct (10-fold dil-undil)** | **Ct undil** | **Ct 10-fold dil** | **∆Ct (10-fold dil-undil)** |
| 1-16S | 21.2 | 24.5 | 3.3 | 20.6 | 24.5 | 3.9 | 22.7 | 25.8 | 3.1 |
| 2-16S | 22.8 | 26.2 | 3.4 | 21.7 | 24.8 | 3.1 | 21.1 | 24.1 | 3.0 |
| 3-16S | 21.3 | 24.5 | 3.2 | 21.4 | 24.4 | 3.0 | 19.6 | 22.8 | 3.2 |
| 4-16S | 20.7 | 23.9 | 3.2 | 21.6 | 25.1 | 3.5 | 18.7 | 21.6 | 2.9 |
| 5-16S | 21.4 | 24.4 | 3.0 | 24.5 | 27.7 | 3.2 | 23.5 | 26.8 | 3.3 |
| 6-16S | 20.7 | 23.8 | 3.1 | 24.7 | 28.0 | 3.3 | 19.8 | 22.6 | 2.8 |
| 7-16S | 18.6 | 21.9 | 3.3 | 22.4 | 25.2 | 2.8 | 19.8 | 23.2 | 3.4 |
| 8-16S | 19.3 | 22.5 | 3.2 | 21.1 | 24.6 | 3.5 | 19.1 | 22 | 2.9 |
| 9-16S | 19.1 | 22.1 | 3.0 | 18.6 | 21.5 | 2.9 | 20.3 | 23.7 | 3.4 |
| 10-16S | 18.2 | 21.1 | 2.9 | 21.3 | 24.8 | 3.5 | 18.9 | 21.8 | 2.9 |
| 11-16S | 18.5 | 21.3 | 2.8 | 24.3 | 27.6 | 3.3 | 19.2 | 22.5 | 3.3 |
| 12-16S | 19.1 | 22.2 | 3.1 | 24.3 | 27.5 | 3.2 | 19.3 | 22.5 | 3.2 |
| 13-16S | 22.2 | 25.6 | 3.4 | 23.1 | 26.5 | 3.4 | 22.5 | 25.3 | 2.8 |
| 14-16S | 21.8 | 25.1 | 3.3 | 21.2 | 24.1 | 2.9 | 23.1 | 26.5 | 3.4 |
| 15-16S | 22.0 | 25.6 | 3.6 | 22.2 | 25.4 | 3.2 | 20.3 | 23.7 | 3.4 |
| 16-16S | 20.5 | 23.8 | 3.3 | 20.3 | 23.7 | 3.4 | 21.1 | 24.6 | 3.5 |
| 17-16S | 20.0 | 23.4 | 3.4 | 23.0 | 26.3 | 3.3 | 21.8 | 25.2 | 3.4 |
| 18-16S | 20.3 | 23.7 | 3.4 | 22.0 | 25.3 | 3.3 | 19.1 | 22.5 | 3.4 |
| 19-16S | 18.4 | 21.5 | 3.1 | 18.5 | 21.2 | **2.7** | 17.7 | 20.2 | **2.5** |
| 20-16S | 18.5 | 22.0 | 3.5 | 21.3 | 24.6 | 3.3 | 19.9 | 23.5 | 3.6 |
| 21-16S | 18.6 | 22.1 | 3.5 | 19.1 | 21.9 | 2.8 | 17.8 | 20.9 | 3.1 |
| 22-16S | 19.4 | 23.0 | 3.6 | 22.5 | 25.7 | 3.2 | 18.6 | 21.7 | 3.1 |
| 23-16S | 20.1 | 23.7 | 3.6 | 20.9 | 24.2 | 3.3 | 20.4 | 24.0 | 3.6 |
| 24-16S | 21.5 | 25.2 | 3.7 | 21.2 | 24.7 | 3.5 | 18.8 | 21.6 | 2.8 |
| 25-16S | 20.9 | 24.4 | 3.5 | 20.8 | 24.6 | 3.8 | 21.6 | 24.5 | 2.9 |
| 26-16S | 20.2 | 23.7 | 3.5 | 21.3 | 24.6 | 3.3 | 22.5 | 26.2 | 3.7 |
| 27-16S | 22.1 | 25.2 | 3.1 | 20.5 | 24.3 | 3.8 | 19.8 | 23.3 | 3.5 |
| 28-16S | 22.1 | 25.8 | 3.7 | 21.9 | 24.2 | **2.3** | 23.3 | 27.1 | 3.8 |
| 29-16S | 21.5 | 25.2 | 3.7 | 19.9 | 24.4 | 4.5 | 21.5 | 25.2 | 3.7 |
| 30-16S | 21.8 | 25.2 | 3.4 | 19.6 | 23.1 | 3.5 | 21.5 | 25.2 | 3.7 |
| 31-16S | 21.0 | 24.2 | 3.2 | 20.0 | 23.6 | 3.6 | 19.3 | 22.4 | 3.1 |
| 32-16S | 19.9 | 23.1 | 3.2 | 19.6 | 22.4 | 2.8 | 18.0 | 21.0 | 3.0 |
| 33-16S | 20.6 | 23.7 | 3.1 | 20.1 | 22.5 | **2.4** | 23.3 | 26.2 | 2.9 |
| 34-16S | 19.6 | 22.5 | 2.9 | 19.6 | 23.1 | 3.5 | 19.9 | 22.4 | **2.5** |
| 35-16S | 20.8 | 23.6 | 2.8 | 20.1 | 23.1 | 3.0 | 19.4 | 22.2 | 2.8 |
| 36-16S | 19.9 | 23.2 | 3.3 | 18.7 | 22.1 | 3.4 | 19.7 | 22.3 | **2.6** |
| No. of inhibited samples |  |  | **0/36** |  |  | **3/36** |  |  | **3/36** |
| 1-GAPDH | 15.8 | 19.1 | 3.3 | 20.6 | 23.6 | 3.0 | 16.2 | 19.1 | 2.9 |
| 2-GAPDH | 15.7 | 19.1 | 3.4 | 19.3 | 23.1 | 3.8 | 16.7 | 20.0 | 3.3 |
| 3-GAPDH | 15 | 18.1 | 3.1 | 19.7 | 23.2 | 3.5 | 16.4 | 19.6 | 3.2 |
| 4-GAPDH | 17.6 | 20.5 | 2.9 | 14 | 17.3 | 3.3 | 17.2 | 20.4 | 3.2 |
| 5-GAPDH | 18.1 | 20.7 | **2.6** | 13.2 | 16.3 | 3.1 | 16.5 | 19.8 | 3.3 |
| 6-GAPDH | 17.9 | 20.7 | 2.8 | 12.7 | 16 | 3.3 | 12.7 | 15.9 | 3.2 |
| 7-GAPDH | 12.6 | 15.7 | 3.1 | 21.4 | 24.6 | 3.2 | 14.4 | 17.7 | 3.3 |
| 8-GAPDH | 13.1 | 16.2 | 3.1 | 18.4 | 21.7 | 3.3 | 11.8 | 15.6 | 3.8 |
| 9-GAPDH | 13.7 | 17.1 | 3.4 | 17 | 19.5 | **2.5** | 12.8 | 16.3 | 3.5 |
| 10-GAPDH | 13.6 | 17.0 | 3.4 | 17.3 | 20.1 | 2.8 | 13.0 | 16.1 | 3.1 |
| 11-GAPDH | 13.8 | 16.7 | 2.9 | 17.9 | 23.3 | 5.4 | 12.9 | 16.2 | 3.3 |
| 12-GAPDH | 13.4 | 16.6 | 3.2 | 19.3 | 22.7 | 3.4 | 15.1 | 18.1 | 3.0 |
| 13-GAPDH | 14.1 | 17.2 | 3.1 | 20.3 | 23.7 | 3.4 | 13.1 | 16.4 | 3.3 |
| 14-GAPDH | 15.1 | 18.4 | 3.3 | 19.1 | 22.3 | 3.2 | 13.4 | 17.0 | 3.6 |
| 15-GAPDH | 13.5 | 17.0 | 3.5 | 18.4 | 21.2 | 2.8 | 14.5 | 17.8 | 3.3 |
| 16-GAPDH | 14.7 | 18.2 | 3.5 | 23.1 | 25.7 | **2.6** | 16.9 | 20.3 | 3.4 |
| 17-GAPDH | 14.5 | 17.9 | 3.4 | 20.5 | 23.9 | 3.4 | 15.9 | 19.5 | 3.6 |
| 18-GAPDH | 13.7 | 17.2 | 3.5 | 19.4 | 22.8 | 3.4 | 14.8 | 18.6 | 3.8 |
| 19-GAPDH | 13.9 | 17.0 | 3.1 | 13.2 | 17.5 | 4.3 | 12.8 | 15.1 | **2.3** |
| 20-GAPDH | 13.2 | 16.6 | 3.4 | 17.4 | 20.6 | 3.2 | 14.9 | 18.4 | 3.5 |
| 21-GAPDH | 12.2 | 15.4 | 3.2 | 11.4 | 15.3 | 3.9 | 13.1 | 16.1 | 3.0 |
| 22-GAPDH | 12.5 | 16.1 | 3.6 | 19.8 | 23.2 | 3.4 | 13.3 | 16.6 | 3.3 |
| 23-GAPDH | 13.4 | 16.9 | 3.5 | 22.1 | 25.5 | 3.4 | 17.2 | 20.7 | 3.5 |
| 24-GAPDH | 17.3 | 20.9 | 3.6 | 18.4 | 21.2 | 2.8 | 13.3 | 16.8 | 3.5 |
| 25-GAPDH | 14.2 | 17.5 | 3.3 | 22.5 | 25.7 | 3.2 | 14.8 | 18.3 | 3.5 |
| 26-GAPDH | 14.3 | 17.7 | 3.4 | 23.3 | 26.4 | 3.1 | 16.4 | 20.2 | 3.8 |
| 27-GAPDH | 14.1 | 17.1 | 3.0 | 21.1 | 24.5 | 3.4 | 16.8 | 20.3 | 3.5 |
| 28-GAPDH | 14.9 | 18.4 | 3.5 | 18.4 | 21.6 | 3.2 | 17.8 | 21.4 | 3.6 |
| 29-GAPDH | 14.6 | 18.2 | 3.6 | 12.6 | 16.3 | 3.7 | 18.2 | 21.8 | 3.6 |
| 30-GAPDH | 15 | 18.3 | 3.3 | 13.6 | 17.2 | 3.6 | 17.2 | 20.9 | 3.7 |
| 31-GAPDH | 14.9 | 17.9 | 3.0 | 14.3 | 17.5 | 3.2 | 12.0 | 15.4 | 3.4 |
| 32-GAPDH | 13.7 | 16.7 | 3.0 | 13.6 | 17.3 | 3.7 | 11.7 | 14.5 | 2.8 |
| 33-GAPDH | 14.3 | 17.2 | 2.9 | 11.2 | 14.9 | 3.7 | 13.4 | 16.8 | 3.4 |
| 34-GAPDH | 13.9 | 17.1 | 3.2 | 15.1 | 18.2 | 3.1 | 12.8 | 15.9 | 3.1 |
| 35-GAPDH | 14 | 17.2 | 3.2 | 13.7 | 17.4 | 3.7 | 12.2 | 15.4 | 3.2 |
| 36-GAPDH | 10.7 | 14.5 | 3.8 | 11.9 | 15.1 | 3.2 | 11.8 | 14.0 | **2.2** |
| No. of inhibited samples |  |  | **1/36** |  |  | **2/36** |  |  | **2/36** |

**References**

Tonetti, M. S., Greenwell, H., & Kornman, K. S. (2018). Staging and grading of periodontitis: Framework and proposal of a new classification and case definition. *Journal of Periodontology*, *89*(S1), S159–S172. https://doi.org/10.1002/JPER.18-0006
